# Drug resistance evolution in patients with human immunodeficiency virus-1 under long-term antiretroviral treatment-failure in Yunnan Province, China

**DOI:** 10.1186/s12985-018-1112-6

**Published:** 2019-01-08

**Authors:** Jianjian Li, Yawen Xu, Jiafa Liu, Bihui Yang, Cuixian Yang, Mi Zhang, Xingqi Dong

**Affiliations:** Yunnan Provincial Hospital of Infectious Disease, AIDS Care Center (YNACC), Kunming Medical University affiliated Infectious diseases hospital, 28 Shian Road, Taiping District, Kunming, 650301 China

**Keywords:** HIV-1, AIDS, Antiretroviral therapeutic failure, Genotyping drug resistance, Evolution

## Abstract

**Background:**

Understanding the prevalence and evolution of HIV-1 drug resistance (DR) and associated mutation patterns is critical to implementing free antiretroviral therapy in Yunnan, the first antiretroviral treatment location in China. Here We provide a basis for understanding the occurrence and development of HIV-1 resistance in Yunnan and a theoretical foundational for strategy to delay HIV-1 drug resistance and achieve successful individualized treatment.

**Methods:**

Plasma samples from different cities/prefectures were collected at Yunnan Provincial Hospital of Infectious Disease from January 2010 to September 2016, and those from drug-resistant individuals were genotyped using in-house assays, 88 patients were selected for the study who had been on treatment for ≥6 months (and for whom drug resistance was then measured), and each patient had at least 3 genotype resistance tests and who were enrolled to analyze mutation and evolution of HIV resistance.

**Results:**

264 Pol sequences of 88 patients were obtained. Drug resistance levels to eight drugs increased to varying degrees with prolonged treatment. Resistance to efavirenz (EFV) and etravirine (ETR) showed the highest change, comparisons of resistant changes to second and first and to third and second agents showed altered level of drug resistance were 25 and 20 cases, 28 and 18 cases, respectively. The smallest change was Lopinavir/Ritonavir (LPV/r) present 2 and 3 cases; Resistance to lamivudine (3TC) and lopinavir/ritonavir (LPV/r) was high among patients detected thrice, whereas other drugs were distributed in all resistance levels. M184 V/I (26.14%), T69S (11.36%), and T215Y/I (10.23%) mutations were the most common in nucleoside reverse transcriptase inhibitors (NRTIs), and K103 N/R/S (21.59%), V179D/E (20.45%) in Non-NRTIs (NNRTIs). Furthermore, L10 V/F/I (6.82%), A71V (4.55%), and I54V (4.55%) mutations were common in protease inhibitors (PIs).

**Conclusions:**

We found dynamic genotypic changes in HIV-1 drug-resistance in Yunnan, with prolonged treatment, and drug resistance was inevitable. However, resistance to different drugs occurred at varying times, and mutation site emergence was the main cause. These findings enhance our understanding of evolution and regulation, and are valuable for developing HIV-1 DR prevention strategies in Yunnan.

## Background

It is estimated that at least 840,000 people are living with human immunodeficiency virus (HIV) in China. This epidemic is characterized by geographical disparities and a higher prevalence among certain subgroups of the population. The number of new cases is approximately 13,000 every month. The Yunnan province is the main area for smuggling illicit drugs into China. Therefore, the occurrence of the first HIV-1 epidemic (caused by subtype B (or B′) in 1989 among intravenous drug users (IDUs) in this province was not a surprise. Currently, the number of infected patients in this province is approximately 100,000, and HIV-1 genotypes B, C, CRF01_AE, CRF07_BC, and CRF08_BC are in circulation.

The Chinese government implemented the National Free Antiretroviral Therapy (ART) Program in 2003, and highly active ARTs (HAARTs) were first provided in Yunnan in 2004. This strategy has markedly improved the morbidity and mortality associated with HIV-1 infection [1–3]. Although ARTs have significantly been beneficial to patients with HIV/acquired immunodeficiency syndrome (AIDS), they have limitations. These include the rapid emergence of drug-resistant strains of HIV after long-term use of HAARTs, under the pressure of selecting antiviral drugs among ARTs [4–6]. Furthermore, suboptimal ART adherence may accelerate the emergence of resistant viruses and compromise ART efficacy in treated patients and transmission recipients [7]. Undoubtedly, drug resistance (DR) has become one of the most extensive limitations of current ARTs [8] and has a major impact on the choice of antiviral drugs. Although a second-line treatment became available through the national program in 2008 [9], after 9 years, DR to this agent has emerged.

The effective prevention of the spread of HIV DR and the control of AIDS progression in already infected individuals is important. However, although numerous studies have investigated the prevalence of drug-resistant HIV variants, most were confined to specific areas or populations in Yunnan [10–13]. Furthermore, many of these studies were cross-sectional investigations, and thus, no comprehensive data are currently available on ART sensitivity levels in DR, and the evolution of mutations was analyzed dynamically. In contrast, our study was cohort-based, participants were followed over time, and DR was assessed multiple times in the same patients.

Our study aimed to conduct a province-wide survey to investigate the occurrence and development of DR to HIV-1 genotype, the extent of changes in drug sensitivity with prolonged treatment, and the appearance and evolution of HIV-1 DR mutant sites. These analyses were performed to understand the factors that may be associated with the onset and progression of DR and to propose ways of minimizing the impact of these factors on the clinical outcome.

## Methods

### Study subjects and sample collection

Blood samples were collected, three or more times, from 100 newly confirmed HIV-1-positive individuals between January 2011 and December 2016. The number of HIV/AIDS patients treated in Yunnan Province during the 6-year period was approximately 60,000, of which 15% (approximately 9000) were treatment failures and 4500 tested positive for DR. We expect that 100 individuals from 11 prefectures represent the larger patient population due to the systematic sampling method to select patients who tested positive for DR more than three times. The HIV-1 infection status was determined using an enzyme-linked immunosorbent assay (ELISA, Wantai, China) and confirmed using a western blot assay (HIV BLOT 2.2, MP Diagnostics, Singapore).

The study population consisted of patients with HIV/AIDS who received HAARTs for more than six months, were under the National Free Antiretroviral Treatment Program (NFATP), which provides lifelong free ARTs to people living with HIV; and met the national treatment criteria [14, 15]. The national treatment criteria were: (1) CD4 cell count ≤0.2 × 10^9^/L, (2) World Health Organization stage III/IV disease, or (3) willingness to receive ARTs regardless of criteria 1 and 2 [16]. Following the standard requirements, the initial first-line drug regimen was two nucleoside reverse transcriptase inhibitors (NRTIs) plus a non-NRTI (NNRTI); generally, the initial treatment plan is AZT/D4T + 3TC + NVP/EFV. Apart from 3TC, any drug to which resistance was developed was replaced with second-line drugs such as TDF and LPV/r.

### Data collection for patient characteristics

Yunnan has AIDS treatment clinics in over 16 prefectures. The patients were regularly followed-up at each time point. At each follow-up, the doctor recorded the patient’s treatment information, including patient characteristics, and uploaded the information onto the province’s therapeutic website, we downloaded and recorded the demographic and epidemiological information of patients from the treatment system, collated the data which contained age**,** marital status, route of transmission, baseline CD4+ count, sample area, and therapeutic regimen. For patients with DR, the treatment and drug replacement information were also recorded.

### HIV-1 RNA extraction and amplification of HIV-1 pol fragments

Plasma was separated from whole blood, and viral RNA was extracted from 500 μL of plasma from HIV-1-positive patients using the Easy Q extraction system (Biomerieux, Lyons, France) according to the manufacturer’s instructions. For amplification of the Pol gene, the first-round amplification was performed using the One Step reverse transcription polymerase chain reaction (RT-PCR, Takara, Dalian, China) with primers for MAW26 (5′-TTGGAAATGTGGAAAGGAAGGAC-3′) and RT21 (5′-CTGTATTTCTGCTATTAAGTCTTTTGATGGG-3′) in a 25-μL reaction volume using 30 cycles. Nested Pol PCR was performed using primers for PRO-1 (5′-CAGAGCCAACAGCCCCACCA-3′) and RT20 (5′-CTGCCAGTTCTAGCTCTGCTTC-3′) for the second round. The cycling conditions used were as described by Li [17]. Positive PCR products were sequenced by the Nuosai Genomics Co. (Beijing, China) with a variety of internal specific primers [18] after purification.

### Editing and DR analysis of pol sequences

All the sequenced fragments were edited and assembled using Sequencher 6.0 DNA sequence analysis software (Gene Codes, Ann Arbor, MI, USA). Clustal W Multiple alignments and manual editing were performed using Bio-Edit 7.0.9.1 software. The obtained sequences were compared to all known sequences in the HIV database using the Basic Local Alignment Search Tool (BLAST, http://hiv-web.lanl.gov/content/sequence/BASIC_BLAST/basic_blast.html) to check for potential contaminations. A phylogenetic tree was constructed by the neighbor-joining method based on Kimura’s two-parameter distance matrix with 1000 bootstrap replicates using Mega 6.0. In the phylogenetic tree, we ensured that there was no cross-contamination. Drug-resistance mutation profiles and antiretroviral susceptibility were assessed using the World Health Organization-recommended list of mutations for DR surveillance.

The nucleotide sequences of the *Pol* gene containing the full-length protease gene and the first 299 codons of the reverse transcriptase (*RT*) gene, were submitted to Stanford University [19, 20] (http://hivdb.stanford.edu/hivdb/by-mutations/). The levels of resistance to commonly used protease and RT inhibitors were analyzed using a genotypic resistance interpretation algorithm. The scores are the sum of each mutation penalty score for each drug. Scores less than 10 indicate susceptibility; scores between 10 and 14 indicate potential low-level resistance; scores between 15 and 29 indicate low-level resistance; scores between 30 and 59 indicate intermediate resistance. Scores of 60 or higher indicate high-level resistance.

### Statistical analysis

Analyses of the 95% confidence interval of the prevalence rates of baseline DR were conducted using the statistical package for the social sciences (SPSS) 19.0 software (SPSS Inc. Chicago, IL, USA). Categorical variables were compared using Fisher’s exact test or a Chi-square test. We determined that patient characteristics were considered statistically significant in the occurrence of DR when *P* < 0.05.

## Results

### Patient demographic characteristics and therapeutic regimens

Between 2011 and 2016, 100 individuals of Yunnan province who had been accepted into the free ART program for more than six months and who had viral loads ≥1000 copies/mL, measured at least three times at a return interval of 1.5 years, were studied.

On at least three separate occasions, genotype resistance tests were carried out on plasma samples from these subjects. After amplification, a genotype test was performed using an in-house method, and the raw sequences were assembled, aligned, and edited. As a result, 264 Pol sequences (the results of three analyses per patient) were obtained. Samples from 12 individuals were not successfully amplified on one or more occasions as these individuals tested negative in all three tests performed to detect PCR products and the obtained sequences of HIV Pol genes. Therefore, we finally included 88 individuals for analysis.

The BLAST search showed no evidence of sample contamination. The demographic characteristics of the participants with the *Pol* gene are summarized in Table 1. The basic demographic information for the 88 subjects was recorded, with the main routes of infection being IDU and heterosexual transmission (40 and 38 cases, 45.45 and 43.18%, respectively).

**Table 1:** The demographic characteristics of 88 HIV/AIDS patients detected genotyping resistance

Characteristics	DR Positive N1 = 54	DR Negative N2 = 34	Total 88	χ^2^	*P*
	No.(%)	No.(%)	No.		
Gender				0.155	0.812
Male	33 (61.1)	23 (67.6)	56		
Female	21 (38.9)	11 (32.4)	32		
Age (years)				5.133^a^	0.162
6–20	5 (9.3)	–	5		
21–40	36 (66.7)	26 (76.5)	62		
41–60	13 (24.0)	7 (20.6)	20		
61–63	–	1 (2.9)	1		
Marital status				0.329	0.828
Unmarried	14 (25.9)	7 (20.6)	21		
Married	25 (46.3)	17 (50.0)	42		
Divorced or widowed	15 (27.8)	10 (29.4)	25		
Route of transmission				7.550^a^	0.11
Blood-born (BB)	2 (3.7)	–	2		
Injection Drug user (IDU)	24(44.4)	16 (47.1)	40		
Heterosexual (HST)	20(37.0)	18 (52.9)	38		
Mother to Children (MTC)	3(5.6)	–	3		
Unknown	5(9.3)	–	5		
Baseline CD4+ count (cells/μl)				17.682^a^	0.001
0–200	27(50.0)	8 (23.5)	35		
201–350	21(38.9)	21 (61.8)	42		
351–500	–	5 (14.7)	5		
undetected	6(11.1)	–	6		
Area				9.069^a^	0.526
Kunming	13(24.1)	9(26.5)	22		
Lincang	8(14.8)	6(17.6)	14		
Honghe	8(14.8)	4(11.8)	12		
Chuxiong	3(5.6)	6(17.6)	9		
Nujiang	3(5.6)	–	3		
Puer	2(3.7)	2(5.9)	4		
Zhaotong	6(11.1)	3(8.8)	9		
Dali	3(5.6)	3(8.8)	6		
Baoshan	2(3.7)	–	2		
Wenshan	4(7.4)	1(2.9)	5		
Yuxi	2(3.7)	–	2		
Therapeutic regimen				11.809^a^	0.107
3TC + NVP + AZT	19(35.2)	21(61.8)	40		
3TC + NVP + D4T	16(29.6)	5(14.7)	21		
EFV + 3TC + AZT	12(22.2)	5(14.7)	17		
EFV + 3TC + D4T	1(1.9)	3(8.8)	4		
EFV + 3TC + TDF	3(5.6)	–	3		
3TC + AZT + Lpv/r	1(1.9)	–	1		
NVP + DDI + D4T	1(1.9)	–	1		
Lpv/r + 3TC + ABC	1(1.9)	–	1		

^a^Fisher’s exact probability Chisquare test. 3TC:Lamivudine, NVP:Nevirapine, D4T:Stavudine, AZT:Zidovudine, EFV:Efavirenz, TDF:Tenofovir disoproxil fumarate Lpv/r:Lopinavir/Ritonavir

Based on the baseline CD4 lymphocyte count, the patients were mainly categorized into two groups: 0–200 cells/mm^3^ (35 cases, 39.77%) and 201–350 cells/mm^3^ (42 cases, 47.73%). Comparatively, the incidence of DR in patients in the 351–500 cells/mm^3^ group was statistically significant.

Most participants lived in Kunming, Lincang, and Honghe. In this study, we enumerated the treatment regimens for 88 individuals when the latest drug-resistance was detected. Patients who appeared to have nevirapine (NVP)-containing regimens had a higher DR incidence, those who had been switched to efavirenz (EFV)-containing regimens had lower rates of resistance, and those on protease inhibitor (PI)-containing regimens were almost completely non-resistant.

### Variations on the extent of genotypic DR

DR variants were identified in 88 of the 100 subjects. The highest proportion of patients with DR detected at any time point was 61.4% (54/88), and the ratios of resistance for the first, second, and third detections were 61.4% (54/88), 59.1% (52/88), and 58.0% (51/88), respectively.

Based on the treatment duration, the degree of resistance to eight antiviral drugs in Yunnan province increased to different extents; there was a high degree of resistance to Lamivudine (3TC) and lopinavir/ritonavir (LPV/r). The mean times at which resistance was assessed using the three DR tests in the 88 patients were 3.49 ± 2.73, 4.49 ± 2.85, and 5.97 ± 2.81 years. Increasing the treatment time also increased the number of DR subjects that received various drugs. Furthermore, the level of DR showed an upward trend, which is described in Table 2.

**Table 2:** Changes of drug sensitivity in 88 treatment-failure HIV/AIDS cases

Drugs/Frequency	Grade of DR(*N*/%)				
	Susceptible	Potential resistance	Low-level resistance	Intermediate resistance	High-level resistance
3TC					
T1	72 (81.82)	–	–	–	16 (18.18)
T2	70 (79.55)	–	–	–	18 (20.45)
T3	69 (78.41)	1 (1.14)	–	–	18 (20.45)
AZT					
T1	83 (94.32)	1 (1.14)	1 (1.14)	–	3 (3.41)
T2	77 (87.50)	3 (3.41)	1 (1.14)	4 (4.55)	3 (3.41)
T3	75 (85.23)	4 (4.55)	2 (2.27)	2 (2.27)	5 (5.68)
D4T					
T1	82 (93.18)	2 (2.27)	–	1 (1.14)	3 (3.41)
T2	75 (85.23)	3 (3.41)	3 (3.41)	4 (4.55)	3 (3.41)
T3	72 (81.82)	4 (4.55)	3 (3.41)	3 (3.41)	6 (6.82)
TDF					
T1	81 (92.05)	–	3 (3.41)	1 (1.14)	3 (3.41)
T2	82 (93.18)	–	2 (2.27)	1 (1.14)	3 (3.41)
T3	78 (88.64)	–	5 (5.68)	2 (2.27)	3 (3.41)
EFV					
T1	51 (57.95)	6 (6.82)	3 (3.41)	8 (9.09)	20 (22.73)
T2	50 (56.82)	6 (6.82)	2 (2.27)	9 (10.23)	21 (23.86)
T3	52 (59.09)	6 (6.82)	1 (1.14)	7 (7.95)	22 (25.00)
ETR					
T1	61 (69.32)	13 (14.77)	6 (6.82)	7 (7.95)	1 (1.14)
T2	58 (65.91)	11 (12.50)	5 (5.68)	10 (11.36)	4 (4.55)
T3	57 (64.77)	13 (14.77)	4 (4.55)	9 (10.23)	5 (5.68)
NVP					
T1	53 (60.23)	5 (5.68)	1 (1.14)	2 (2.27)	27 (30.68)
T2	51 (57.95)	5 (5.68)	1 (1.14)	2 (2.27)	29 (32.95)
T3	51 (57.95)	6 (6.82)	1 (1.14)	1 (1.14)	29 (32.95)
LPV/r					
T1	87 (98.86)	–	–	–	1 (1.14)
T2	85 (96.59)	–	–	1 (1.14)	2 (2.27)
T3	84 (95.45)	–	–	1 (1.14)	2 (2.27)

T1: First detection, T2, T3 and the like

### DR variation in treatment-failed individuals

Next, we evaluated the changes in DR in the 88 treatment-failed individuals by comparing their second and first, and third and second results. The changes in DR among different patients are summarized in Table 3. Most cases with DR changes occurred following ETR, EFV, and NVP. Conversely, the least number of resistance changes occurred following LPV/r and TDF. Treatment with NVP had the highest number of drug-resistant degree decreased 4 grades and treatment with 3TC had the highest number of drug-resistant degree increased 4 grades.

**Table 3:** The dynamic variability of drug susceptibility in 88 HIV/AIDS patients

Drugs	Numbers of drug susceptibility undergo changes									Total (*N*/%)
	−4	−3	−2	−1	invariant	1	2	3	4	
3TC										
T2 vs T1	4	0	0	0	77	0	0	0	7	11 (12.50)
T3 vs T2	8	0	0	0	74	1	0	0	5	14 (15.91)
AZT										
T2 vs T1	0	0	1	1	79	1	1	3	2	9 (10.23)
T3 vs T2	1	1	1	0	81	2	1	0	1	7 (7.95)
D4T										
T2 vs T1	0	0	0	3	75	3	3	2	2	13 (14.77)
T3 vs T2	2	2	0	0	75	5	2	1	1	13 (14.77)
TDF										
T2 vs T1	0	0	0	2	82	1	1	1	1	6 (6.82)
T3 vs T2	1	1	1	0	80	1	2	1	1	8 (9.09)
EFV										
T2 vs T1	4	4	1	3	63	4	1	4	4	25 (28.41)
T3 vs T2	3	4	0	3	68	5	1	0	4	20 (22.73)
ETR										
T2 vs T1	0	2	1	6	60	9	5	5	0	28 (31.82)
T3 vs T2	0	4	1	3	70	7	1	2	0	18 (20.45)
NVP										
T2 vs T1	6	2	0	3	67	0	3	0	7	21 (23.86)
T3 vs T2	6	0	0	2	71	3	1	1	4	17 (19.32)
LPV/r										
T2 vs T1	0	0	0	0	86	0	0	1	1	2 (2.27)
T3 vs T2	1	0	0	0	85	1	0	1	0	3 (3.41)

T2 vs T1: Comparison of second detection and first detection, T3 vs T2: Comparison of third detection and second detection

−4 represent drug resistant degree reduce 4 grades (For example, the first detected result was high level resistance, the second result change to Susceptible), opposite 4 represent drug resistant degree raise 4 grades, the others and the like

### Occurrence of NRTI, NNRTI, and PI mutation sites in treatment-failed participants who were identified as having DR three times

For the 88 HIV cases with Pol sequences, 61 were positive for the tested mutations. New mutations can emerge in the same patient as treatment progresses; however, early mutations can also disappear with drug administration.

The most common NRTIs mutations were M184 V/I, T69S, and T215Y/I, with DR rates of 37.7, 16.39, and 14.75%, respectively. The mutation M184 V/I principally appeared in the first antiviral treatment failure, whereas the T215Y/I mutation was mainly associated with the second and third occasions.

The most common NNRTIs mutations were K103 N/R/S, V179D/E, and G190A, with DR rates of 21.59, 20.45, and 18.18%, respectively. The NNRTI-related mutations mainly appeared in the first and second tests.

**Fig. 1:**
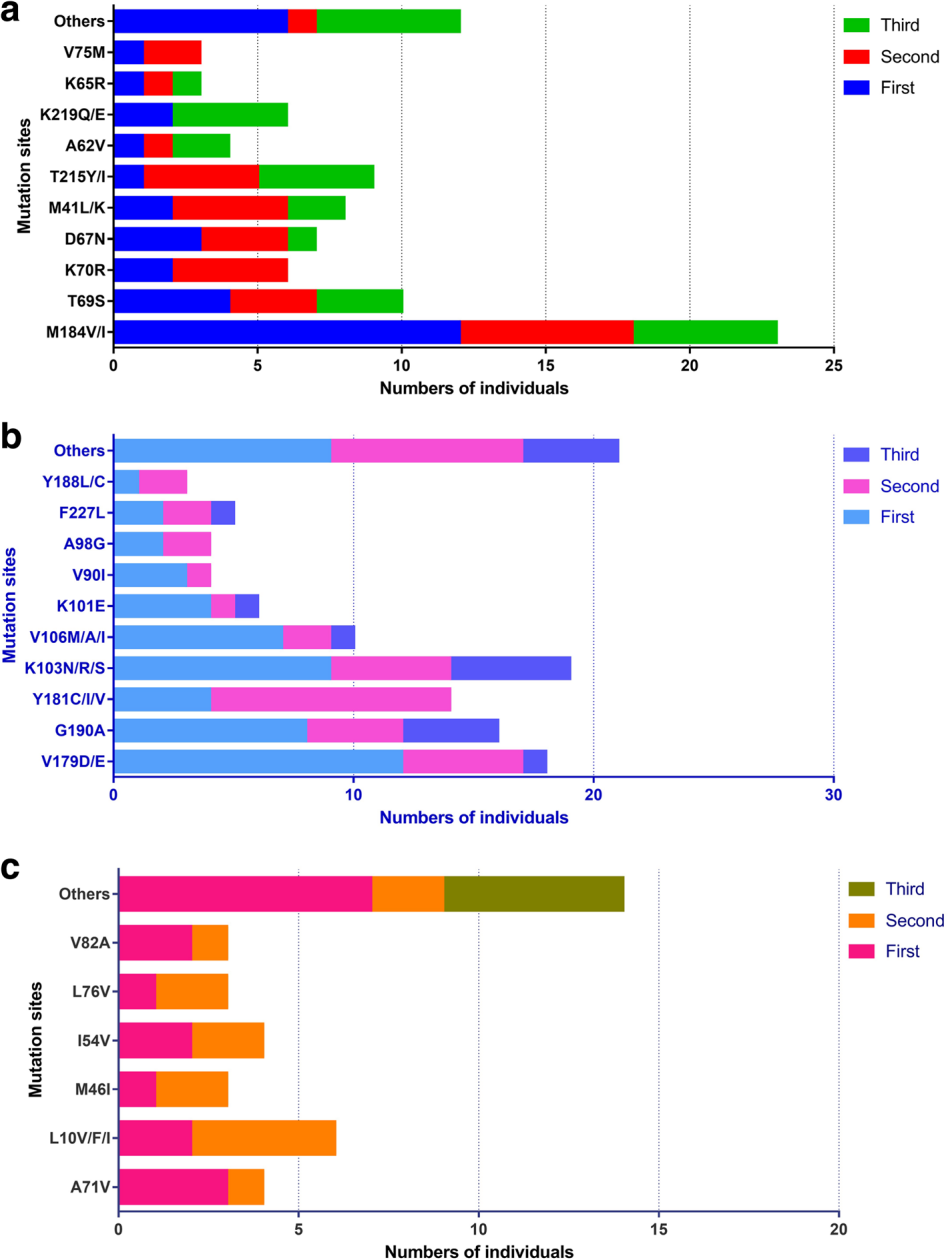
Prevalence of resistant mutations for three kinds ART drugs in long-term treatment HIV infected individuals residing in Yunnan. **a**-**c** was drawed represent NRTIs, NNRTIs and PIs, respectively. The X axis indicates the number of individuals with DR mutations. The first, second and third test results presented in the figures were marked separately in different colour

The PI mutations most commonly found were L10 V/F/I, A71V, and I54V, with DR rates of 6.82, 4.55, and 4.55%, respectively. These latter sites were affected in the early stages of treatment. The mutations associated with DR in patients with ART failure are illustrated in Fig. 1.

## Discussion

In this study, we conducted a cohort analysis, using broad surveillance of HIV drug-resistance. One hundred HIV-1-positive individuals from 11 prefectures were enrolled in the study and 88 were subjected to DR analysis. Since all participants were from Yunnan, our data are not representative of the overall situation of drug-resistant variants. However, these data on the prevalence of drug-resistant variants will have important implications for clinical therapy, concerning genetic evolution.

Since ART was made available free of cost across Yunnan, a significant number of individuals infected through sexual transmission accepted the free treatment [21]. Moreover, an argument could be made that the spread of HIV drug-resistance would likely be higher among ART individuals because the therapy has been administered for an extended period in this area. As a result, resistant strains were detected in 61% of individuals in which ART failed, implying that this high prevalence closely correlated with the widespread use of ART drugs.

Our study shows that ART duration positively correlated with an increased incidence of HIV-1 drug-resistant strains. In other words, the longer the duration of ART, the higher the probability of developing drug-resistance. Therefore, it is important to elucidate the occurrence and development of HIV DR as well as changes in mutations in Yunnan.

Seven factors were taken into account in the DR and non-DR groups to determine the risk factors associated with HIV-1 DR. We inittially thought that the high prevalence of HIV DR among individuals infected through IDU was due to better adherence in individuals infected through sexual transmission, resulting in lower HIV DR [22–24]. However, there were no differences in DR occurrence among the two causes of transmission.

Interestingly, when we analyzed the association between HIV DR and CD4+ counts, we found a statistical difference between the group with < 200 cells/μL and the group with 351–500 cells/μL, showing a close correlation between cell count and the development of drug-resistant strains, in addition to having a significant impact on the risk of mortality [25]. This supports that the lower the CD4 + cell count, the higher the risk of DR emergence. In other words, the lower the CD4 + T lymphocyte count, the more difficult it is to recover the immune system. Therefore, it is advantageous to start medication early in these patients to keep the immune system less compromised even if this possibly increases the DR onset.

Zaccaerlli [26] compared the findings of historical and current tests for resistance over long-term ART; he found that the tolerance after three types of antiviral drugs based on the historical test was 25% higher than that based on the final test; this outcome was also observed in Punthiya’s [27] study. In the present study, however, the susceptibility to the three types of drugs tended to decrease throughout the antiviral treatment (except for EFV in Yunnan province). This indicates that the longer the antiviral therapy, the higher the possibility of DR, thus making resistance, in addition to adverse drug reactions and patient adherence, an inevitable outcome of long-term treatment.

However, EFV was an exception to the trend as susceptibility in the third test was higher than that of the previous two tests. We surmise that the dose requirement of EFV in China is relatively high, resulting in a higher blood concentration, which slows the conversion of the virus into a resistant strain. For low-level and moderate DR, the resistance to the three types of drugs increased with the duration of treatment, but the opposite trend was observed for EFV. We highlight that the DR surveillance network at Stanford University is likely to use a mutation at a single locus to provide a definitive standard for interpreting EFV resistance results; this may have affected our results.

The rates of high-level resistance were elevated for all the drugs assessed, which coincided with a decrease in the sensitivity rates. From the dynamic changes in drug susceptibility, we demonstrated that the extent changes of apparent DR were the highest for EFV, followed by NVP, and 3TC. As the duration of the therapy increased, the susceptibility to these three drugs decreased markedly, adherence in some patients was also reduced with prolonged treatment time, causing an increase in the number of resistant patients. However, the rise in drug susceptibility might be because ART medical professionals found, based on the initial DR reports, that the drugs were losing efficacy, and therefore, discontinued them, resulting in the disappearance of drug stress.

Mutation at sites such as M184 V/I and K103 N are important because they cause resistance to several drugs, and thus, play a decisive role in the extent to which DR changes over time. Some patients who were initially susceptible transitioned to potentially drug-resistant at the second analysis. As time progressed, they developed low-level, moderate, or high-level resistance. This potential resistance should be treated as a red-flag warning of the need to improve adherence or switch to replacement drugs and must be swiftly acted upon to avoid the development of higher degrees of DR.

The interaction between the host, drug, and virus contributes to DR in HIV because the virus develops strategies to escape drug pressure. For example, it mutates self-resistant gene sites to resist the therapeutic effect of ART drugs. Antiviral treatment failure in patients with HIV appears inevitable. Thus, timely detection of genotypic susceptibility to DR and an adjustment of the treatment regimen might improve ART success.

Some of the most common nucleoside analog-associated mutations (NAMs) are the thymidine-associated mutations (TAMs). Two clusters were studied here; TAM-1 (M41 L and T215Y) and TAM-2 (D67N, K70R, and K219Q). The viruses that present TAM-1 s usually produce more cross-resistance to other NRTIs, whereas the cross-resistance of the viruses carrying TAM-2 is very low. The resistance rates of patients carrying the AZT and D4T mutations increased at all stages; this may be due to the long time that TAMs take to develop.

M184 V/I was the most frequent NRTI mutation found during the first and second analyses. Another study showed that when 3TC was used as a single drug, M184 V/I resistant strains replaced wild strains in a few weeks and that when 3TC was used in combination treatments, M184 V/I DR strains always appeared first [28]. This may explain the high resistance caused by and the incidence of the M184 V/I mutation observed in this study when 3TC was used.

Within the three major classes of antiviral drugs, NNRTIs are the most complex, having many mutations. We observed that NNRTI mutations principally occurred in the first and second periods of treatment failure and were mainly K103 N/R/S, V179D/E, Y181C/I/V, G190A, Y188L/C. Because these mutations arose much earlier in the course of treatment, leading to high resistance to one or more NNRTIs, we deduce that mutation at these sites has an impact on the DR to all NNRTIs. Even though a NNRTI could replace another after failure, these mutations persisted.

Previous studies have performed adaptive sequencing of subjects receiving NNRTIs, [29, 30] and confirmed that the most common mutations are as follows: Y181C/I/V > K103 N/R/S > G190A > V106A. In this study, the occurrence rates for K103 N/R/S, G190A, and V106A were higher than that for Y181C/I/V; however, after a long period, the Y181C/I/V and K103 N/R/S mutations showed a greater prevalence. A competitive relationship among drug-resistant strains during long-term antiviral treatment (especially with NNRTIs) exists due to the presence of Y181C/I/V and K103 N/R/S, which affect the binding of NNRTIs to the active sites of the enzymes.

As PIs are not widely used in patients with HIV/AIDS in and around our province, the frequencies of major or minor DR-related mutations were very low. L10 V/F/I had the highest rate (up to 6.82%). None of the major PI mutations emerged during the third monitoring. We believe that the occurrence of these mutations does not lead to real DR; the observed DR would likely be due to the transmitted strains.

We acknowledge the limitations of this study. Most of these patients came from rural areas or towns and villages where the antiviral treatment and management could be improved, and the medical staff capacity is limited. Thus, it is possible that many of these patients were not switched to the second-line drugs when resistance to the first-line drugs occurred or did not completely follow the National Antiviral Treatment Manual Standards to replace the drugs. Therefore, we cannot accurately account for the drug replacement process for these 88 individuals. This is the major drawback of this study.

## Conclusion

The prevalence of HIV-1 DR in individuals with long-term ART-failure in Yunnan Province is likely the cause of the current hyper-endemic state. Based on our results, DR appears to be an inevitable outcome; however, we have found some evolutionary rules underlying its development and progression. Developing strategies to effectively optimize regimens in local regions and promote adherence to long-term medication will be an arduous task but will be important and of great significance to improve the control and prevention of AIDS in this area.

## Abbreviations

3TC: Lamivudine
ABC: Abacavir
AIDS: Acquire Immune Deficiency Syndrome
AZT: Zidovudine
D4T: Stavudine
DDI: Didanosine
EFV: Efavirenz
ETR: Etravirine
FTC: Emtricitabine
HAART: Highly Active Antiretrovirus therapy
HIV-1: Human Immunodeficiency Virus type
LPV/r: Lopinavir+Ritonavir
NNRTIs: Non-nucleoside analogue reverse transcriptase inhibitors
NRTIs: Nucleoside analogue reverse transcriptase inhibitors
NVP: Nevirapine
PIs: Protease inhibitors
RPV: Rilpivirine
TAMs: Thymidine analog mutations
TDF: Tenofovir disoproxil fumarate

## References

[1] Broder S. The development of antiretroviral therapy and its impact on the HIV-1/AIDS pandemic. Antivir Res. 2010;85:1–18. 10.1016/j.antiviral.2009.10.002 PMID: 20018391. 10.1016/j.antiviral.2009.10.002 PMC2815149 20018391

[2] Tsibris AM, Hirsch MS. Antiretroviral therapy in the clinic. J Virol. 2010;84:5458–64. 10.1128/JVI.02524-09 PMID: 20181709. 10.1128/JVI.02524-09 PMC2876604 20181709

[3] Volberding PA, Deeks SG (2010). Antiretroviral therapy and management of HIV infection. Lancet.

[4] Zhang KL, Zhou JS (2011). Recapturing public health sciences for HIV/AIDS in China. Public Health.

[5] Zhao Y, Mu W, Harwell J, Zhou H, Sun X, Cheng Y (2011). Drug resistance profiles among HIV-1-infected children experiencing delayed switch and 12-month efficacy after using second-line antiretroviral therapy: an observational cohort study in rural China. J Acquir Immune Defic Syndr.

[6] Li JY, Li HP, Li L, Li H, Wang Z, Yang K, et al. Prevalence and evolution of drug resistance HIV-1 variants in Henan. China Cell Res. 2005;15(11–12):843–9. 10.1038/sj.cr.7290356 PMID: 16354557. 10.1038/sj.cr.7290356 16354557

[7] Jose L (2015). Moreno, Delwyn C, Hyoung S, Lee, Kathy G. the relationship between ART adherence and smoking status among HIV+ individuals. AIDS Behav.

[8] Paredes R, Clotet B (2010). Clinical management of HIV-1 resistance. Antivir Res.

[9] Luo L, Li TS. Overview of antiretroviral treatment in China: advancement and challenge. Chin Med J.2011(Engl); 124(3): 440–444. PMID: 21362348 21362348

[10] Su Q, Liang H, Cen P, Bi Z, Zhou P. HIV type 1 subtypes based on the pol gene and drug resistance mutations among antiretroviralnaive patients from Guangxi, Southern China. AIDS Res Hum Retrovir. 2012;28(7):725–8 10.1089/AID.2011.0246. PMID: 21916807. 10.1089/AID.2011.0246 21916807

[11] Ma L, Huang J, Xing H, Yuan L, Yu X, Sun J (2010). Genotypic and phenotypic cross-drug resistance of harboring drug-resistant HIV type 1 subtype B' strains from former blood donors in central Chinese provinces. AIDS Res Hum Retrovir.

[12] Wang X, He C, Xing H, Liao L, Xu X, He J (2012). Short communication: emerging transmitted HIV type 1 drug resistance mutations among patients prior to start of first-line antiretroviral therapy in middle and low prevalence sites in China. AIDS Res Hum Retrovir.

[13] Zhao B, Han X, Dai D, Liu J, Ding H, Xu J, et al. New trends of primary drug resistance among HIV type 1-infected men who have sex with men in Liaoning Province, China. AIDS Res Hum Retrovir. 2011;27(10):1047–53 10.1089/AID.2010.0119. PMID: 21417755. 10.1089/AID.2010.0119 21417755

[14] Wang X, Yang L, Li H, Zuo L, Liang S, Liu W (2011). Factors associated with HIV virologic failure among patients on HAART for one year at three sentinel surveillance sites in China. Curr HIV Res.

[15] Zhang F, Dou Z, Ma Y, Zhao Y, Liu Z, Bulterys M (2009). Five-year outcomes of the China National Free Antiretroviral Treatment Program. Ann Intern Med.

[16] Chinese Center of Disease and Control. Manual of the national free antiretroviral treatment, 2007.

[17] Li L, Lu X, Li H, Chen L, Wang Z, Liu Y (2011). High genetic diversity of HIV-1 was found in men who have sex with men in Shijiazhuang. China Infect Genet Evol.

[18] Li L, Liang S, Chen L, Liu W, Li H, Liu Y (2010). Genetic characterization of 13 subtype CRF01_AE near full-length genomes in Guangxi. China. AIDS Res Hum Retrovir..

[19] Bennett DE, Camacho RJ, Otelea D, Kuritzkes DR, Fleury H, Kiuchi M, et al. Drug resistance mutations for surveillance of transmitted HIV-1 drug-resistance: 2009 update. PLoS One. 2009;4(3):e4724 10.1371/journal.pone.0004724. PMID:19266092. 10.1371/journal.pone.0004724 PMC2648874 19266092

[20] Shafer RW, Rhee SY, Pillay D, Miller V, Sandstrom P, Schapiro JM (2007). HIV-1 protease and reverse transcriptase mutations for drug resistance surveillance. AIDS.

[21] Yuan Y, Liu H, Liu C, Wang Z, Ruan Y, Xing H (2016). An analysis of HIV-1 mutations conferring drug resistance in non-responders to highly active antiretroviral therapy (HAART) in Central Henan province. J Pathog Biol.

[22] Xing Y, Sun J, Cao W, Lee L, Guo H, Li H (2012). Economic evaluation of methadone maintenance treatment in HIV/AIDS control among injecting drug users in Dehong, China. AIDS Care.

[23] Yang F, Lin P, Li Y, He Q, Long Q, Fu X (2013). Predictors of retention in community-based methadone maintenance treatment program in Pearl River Delta, China. Harm Reduct J.

[24] Xiao L, Wu Z, Luo W, Wei X. Quality of life of outpatients in methadone maintenance treatment clinics. J Acquir Immune Defic Syndr Suppl 1. 2010: S116–120. 10.1097/QAI.0b013e3181c7dfb5. PMID: 20104102 10.1097/QAI.0b013e3181c7dfb5 PMC2818825 20104102

[25] Mills EJ, Bakanda C, Birungi J, Yaya S, Ford N, et al. The prognostic value of baseline CD4(+) cell count beyond 6 months of antiretroviral therapy in HIV positive individuals in a resource-limited setting. AIDS. 2012;6:1425–9 10.1097/QAD.0b013e328354bf43. PMID: 22526520. 10.1097/QAD.0b013e328354bf43 22526520

[26] Zaccarelli M, Lorenzini P, Ceccherini-Sillberstein F, Tozzi V, Forbici F, Gori C (2009). Historical resistance profile helps to predict salvage failure. Antivir Ther.

[27] Punthiya P, Nareenart I, Wasun C, Sukasem C, Sungkanuparph S (2012). HIV drug resistance interpreted by cumulative versus last Genotypies in HIV-infected patients with multiple Treatmen failures. Current HIV Reasearch.

[28] Wensing AM, Calvez V, Günthard HF, Johnson VA, Paredes R, Pillay D (2015). 2015 update of the drug resistance mutations in HIV-1. Top Antivir Med.

[29] Jiong W, Robert A, Lisa M, Carrie D (2010). Reduced Fitness in Cell Culture of HIV-1 with Nonnucleoside Reverse Transcriptase Inhibitor-Resistant Mutations Correlates with Relative Levels of Reverse Transcriptase Content and RNase H Activity in Virions. J Virol.

[30] Xu HT, Oliveira M, Quan YJ, Bar-Magen T, Wainberg MA (2010). Differential impact of the HIV-1non-nucleoside reverse transcriptase inhibitor mutations K103N and M230L on viral replication and enzyme function. J Antimicrob Chemother.

